# Reactive Postural Responses to Continuous Yaw Perturbations in Healthy Humans: The Effect of Aging

**DOI:** 10.3390/s20010063

**Published:** 2019-12-20

**Authors:** Ilaria Mileti, Juri Taborri, Stefano Rossi, Zaccaria Del Prete, Marco Paoloni, Antonio Suppa, Eduardo Palermo

**Affiliations:** 1 Department of Mechanical and Aerospace Engineering, Sapienza University of Rome, 00184 Rome, Italy; zaccaria.delprete@uniroma1.it (Z.D.P.); eduardo.palermo@uniroma1.it (E.P.); 2 Department of Economics, Engineering, Society and Business Organization (DEIM), University of Tuscia, 01100 Viterbo, Italy; juri.taborri@unitus.it (J.T.); stefano.rossi@unitus.it (S.R.); 3 Department of Physical Medicine and Rehabilitation, Sapienza University of Rome, 00185 Rome, Italy; marco.paoloni@uniroma1.it; 4 Department of Human Neurosciences, Sapienza University of Rome, 00185 Rome, Italy; antonio.suppa@uniroma1.it; 5 IRCCS Neuromed Institute, 86077 Pozzilli (IS), Italy

**Keywords:** aging, reactive postural responses, yaw perturbation, kinematics, postural stability, dynamic posturography

## Abstract

Maintaining balance stability while turning in a quasi-static stance and/or in dynamic motion requires proper recovery mechanisms to manage sudden center-of-mass displacement. Furthermore, falls during turning are among the main concerns of community-dwelling elderly population. This study investigates the effect of aging on reactive postural responses to continuous yaw perturbations on a cohort of 10 young adults (mean age 28 ± 3 years old) and 10 older adults (mean age 61 ± 4 years old). Subjects underwent external continuous yaw perturbations provided by the RotoBit^1D^ platform. Different conditions of visual feedback (eyes opened and eyes closed) and perturbation intensity, i.e., sinusoidal rotations on the horizontal plane at different frequencies (0.2 Hz and 0.3 Hz), were applied. Kinematics of axial body segments was gathered using three inertial measurement units. In order to measure reactive postural responses, we measured body-absolute and joint absolute rotations, center-of-mass displacement, body sway, and inter-joint coordination. Older adults showed significant reduction in horizontal rotations of body segments and joints, as well as in center-of-mass displacement. Furthermore, older adults manifested a greater variability in reactive postural responses than younger adults. The abnormal reactive postural responses observed in older adults might contribute to the well-known age-related difficulty in dealing with balance control during turning.

## 1. Introduction

Falls are among the most common leading causes of accidental death, hospitalization, or injuries, such as broken bones, head, and spinal cord injuries [1]. Since falling still represents a challenging social, medical, and economical matter [2], several research efforts have been spent in understanding the mechanisms leading to falls in the elderly [3]. A possible theory for explaining the increased risk of falling in the elderly population is that age compromises balance capability. However, understanding the effects of aging on balance is still an unanswered question.

Exploring dynamic balance control of upright stance through imposed external perturbations has the main objective to identify deficit in reactive postural responses. According to the amplitude of COM dislocation and the intensity of perturbation, different reactive strategies, such as hip and ankle strategies and/or stepping responses, prevent imbalance [4,5].

Various imposed posture manipulations, such as mechanical, visual, and vestibular perturbations, have been designed to reproduce free-living imbalance conditions and to investigate risk of fall [6,7,8,9], especially in elderly [10,11,12,13,14]. In a mechanical perspective, popular approaches focused on rotational [8,13,15,16,17] and/or translational support surface movements [9,18,19] or on body release paradigms [20,21] to simulate common postural disturbances (e.g., standing on a bus, slipping on a slippery surface, and falling due to a sudden boost). In this context, the majority of the literature studies focused on the postural responses under forward/backward perturbations to assess risk of fall in the elderly. Across this range of perturbation paradigms, older adults exhibited greater difficulty than young adults in recovering loss of balance through protective postural strategies, such as stepping, especially in case of a forward fall. Accordingly, a lower maximum body lean angle [20], smaller peak knee extensor torques, and larger peak extensors torques at hip and ankle joints [21] were reported in older adults during step response in forward perturbations. Conversely, when considering backward perturbations, older adults showed shorter reaction times than during forward perturbations to avoid fall [22].

Age-related postural deficits were also investigated considering different perturbation paradigms such as sinusoidal translating perturbation in the anterior-posterior direction [14], random rotations around sagittal axis [17], slippery floor surface [23], and mixed forward/backward and left/right platform translations [24]. More specifically, Nardone et al. found a greater head stabilization strategy and a looser coupling between head and hip motion in the eyes opened condition in older adults [14]. While, in the study of Cenciarini et al., older adults presented significantly higher active stiffness as compared to young adults to maintain body balance and to counteract destabilizations effects [17]. Moreover, impaired motor patterns in older adults have been also linked to an abnormal postural sway under forward/backward and left/right perturbation directions as attested in the study of Liaw et al. [24].

Although much has been learned about postural responses of older adults exposed to the before mentioned perturbation paradigms, the motor strategies involved in maintaining balance under perturbations around the vertical axis are still unclear. In healthy subjects, reactive postural responses during rotational perturbations around the vertical axis imposed by a robotic platform have been investigated only by few studies [10,11,15]. Earlier postural responses of the distal compared to the proximal body segment were observed both in kinematics [10] and in muscle activity [11]. The evaluation of reactive postural responses in term of COM displacement control, coordination of body segments and body sway response during this specific rotation would offer an ecological analysis of fall-inducing factors in everyday life. In fact, in the elderly, falls frequently occur within a narrowing familiar environment, such as the kitchen and the bathroom, during activities required for basic mobility, such as turning in place [25].

Over the last decade, measuring motor impairments through portable and wearable devices have demonstrated to be of great impact in managing neuromotor and aging deficits [26,27,28]. In fact, the several advantages of wearable sensors, such as the low cost, the high portability, the limited size and the ease-of-use, encourage the use of these technologies in clinical setting as a useful tool for monitoring assessment [29,30]. In this context, the aim of this study is to investigate the effects of aging on reactive postural responses to rotational perturbations around the vertical axis, by using a rotating platform and wearable inertial sensors in different visual conditions. Clarifying changes of dynamic postural control in older adults during rotational perturbations around the vertical axis would help to understand the mechanisms leading to falls when turning. This in turn, would be useful for the design of effective strategies for falls prevention.

## 2. Materials and Methods

### 2.1. Subjects

A cohort of ten healthy older adults (six females and four males, mean age 61 ± 4 years old, mean body mass 68 ± 9 kg, and mean height 170 ± 6 cm) and a cohort of ten young adults (five females and five males, mean age 28 ± 3 years old, mean body mass 63 ± 14 kg, and mean height 169 ± 11 cm) were enrolled. All participants were community-dwelling, medically stable, and able to walk and stand independently without aids. Subjects with intellectual, vestibular and/or visual deficits, neuromuscular diseases, orthopedic and/or neurological surgery interventions in the last three years were excluded from this study. All participants gave written consent before being included in the experimental session. The protocol was designed and conducted in accordance with the Ethical Standard of the 1964 Declaration of Helsinki.

### 2.2. Experimental Setup

The RotoBit^1D^ was used in this study to provide sinusoidal perturbations around the vertical axis [15]. It is a rigid, round, flat robotic platform with a diameter of 0.5 m that allows a comfortable upright bipedal stance without narrowing feet. The mechanical design consists of a servo motor (SANYO DENKI) with maximum torque of 1.96 Nm, an incremental encoder, a toothed belt (PowerGrip HDT), speed reducer, and a polyethylene rotating disk. The robotic platform was computer-controlled by an ad-hoc LabVIEW software program (v.2014, National Instruments, Austin, TX, USA). Two sinusoidal perturbations around vertical axis were designed with fixed peak amplitude of ± 55° C and frequencies of (i) 0.2 Hz and (ii) 0.3 Hz, namely lower (L) and higher (H) frequency, respectively. The peak angular velocity was 80 °C/s and 100 °C/s for the lower and higher frequency, respectively. To avoid sudden variation in starting/stopping velocity, a sigmoidal wave was added at the start/end of the sinusoidal trajectory. In pilot trials, we chose rotation parameters as those able to provide a high perturbation intensity without requiring stepping response of subjects. Inertial Measurement Units (IMUs) (MTw, Xsens Technologies—NL) including a 3-axes accelerometer (± 160 m/s^2^ FS), a 3-axes gyroscope (± 1200 °C/s FS), and a 3-axes magnetometer (± 1.5 Gauss FS) were used for gathering kinematic data of pelvis, trunk, and head. More specifically, the pelvis-sensor was placed centered on the median sacral crest and just below the anterior sacral promontory. The trunk-sensor was placed under the suprasternal notch on the sternum body; while, the head-sensor was placed on the frontal bone over the superciliary arch. Each subject was instrumented by the same expert operator, to guarantee consistent sensor location on body segment.

IMUs were placed by means of suitable elastic belts to avoid relative movement between sensor and body. Sampling frequency was set at 40 Hz. Equipment was simultaneously triggered at both the beginning and the end of each acquisition. More specifically, the ad-hoc LabVIEW software was designed to simultaneously drive the RotoBit1D servo motor and provide an external trigger, i.e., a square signal ranging from 0 to +5 V, to the IMUs Awinda Station through an NI USB-6212 DAQ board. The Awinda was set to start and end the IMUs’ acquisition on the rising and the falling edge of the trigger signal, respectively.

### 2.3. Experimental Procedure

The experimental protocol was conducted at the Department of Physical Medicine and Rehabilitation, Sapienza University of Rome, Italy. Before each session, all tested subjects were asked to perform a Functional Calibration procedure advised by an operator. The FC procedure provided sensor orientations with respect to body segment, to complete the body-to-sensor alignment procedure [31]. The FC procedure consisted of a standing and sitting task, each lasting 5 s. Afterwards, all subjects stood in a comfortable upright bipedal position with vertically hanging arms and externally rotated feet at a preferred angle with a symmetrical placement, on the top center of the robotic platform. All subjects were asked to wear heelless shoes. The experimental procedure included two different perturbation frequencies (lower and higher frequency) and two different visual conditions (eyes opened (EO) and eyes closed (EC)). More specifically, the participants’ reactive postural responses were measured under four balance tasks: standing with EO during platform rotation at (i) lower (EO-L) and (ii) higher (EO-H) frequencies; and standing with EC considering both (iii) lower (EC-L) and (iv) higher (EC-H) platform frequency rotations. In the EO condition, subjects were asked to stare at a fixed point placed on the wall at 2 m from the platform.

Each task was performed three times randomizing the task order across subjects to avoid bias in results due to similar task sequences. In addition, subjects were not advised about frequency, to avoid habituation of postural responses or anticipatory strategies due to predictability [32].

### 2.4. Data Analysis

All data were analyzed off-line using MATLAB (v.2015b, MathWorks, Natick, MA) program. Angular rotations around the vertical axis, i.e., yaw angles, of the platform (pt), the head (h), the trunk (t), and the pelvis (p) segments were considered for the data analysis. Comparisons between rotation of the platform and body segment rotations in the transversal plane were obtained via the fast Fourier transform analysis, by considering Gain ratio (G) and phase shift (φ) indices, akin to [15]. G was computed as the ratio between the maximum amplitude value of the fundamental wave of the first signal and the amplitude value of the second signal at the same frequency. φ was obtained as the difference of the phase angles of the Fourier transform of the two signals at the frequencies having the maximum amplitude in the Fourier domain. Before transforming in Fourier domain, each body segment rotation in the transversal plane was demeaned. Among angular rotation around the vertical axis of the three body segments and platform, 6 pairs were considered for the analysis: platform-head, platform-trunk, platform-pelvis, head-trunk, trunk-pelvis, and head-pelvis. The following nomenclatures were chosen for G and φ indices to indicate the 6 pairs:

(1) $${}^{f}G_{s},\;{}^{f}\varphi_{s}$$

where f and s represent the first and second sine waves considered, respectively. Perfect agreement in amplitude and timing between the first and the second element of the pair were observed considering a G value close to 100% and a φ value close to 0 °C. Instead, an anticipation/delay in phase angle of the second sine wave compared to the first one was defined as a positive/negative phase shift. For sake of clearness, ${}^{pt}G_{h}$, ${}^{pt}G_{t}$, ${}^{pt}G_{p}$, ${}^{pt}\varphi_{h}$, ${}^{pt}\varphi_{t}$, and ${}^{pt}\varphi_{p}$ were addressed as G-absolute and φ-absolute, because each of the yaw body angles (second sine wave) is referred to the yaw platform angle (first sine wave). While, ${}^{h}G_{t}$, ${}^{t}G_{p}$, ${}^{h}G_{p}$, ${}^{h}\varphi_{t}$, ${}^{t}\varphi_{p}$, and ${}^{h}\varphi_{p}$ were addressed as G-relative and φ-relative, because both the first and the second sine wave are referred to a body segment sine wave.

To assess inter-joint coordination on the transversal plane, the continuous relative phase (CRP) technique was computed on yaw body angles, i.e., body rotation on transversal plane [33]. The CRP analyzes the differences in phase angles of two body segments during a particular motion task. Differently from φ index, which provides the average phase shift of the specific sine wave above described, the CRP refers to all the frequency components of a signal, reporting coupling behaviors of two body segments over the entire motor task. Concerning the CRP analyses, the phase space usually consists of the time-dependent measured signal and its first derivative. Thus, the CRP for a particular task is obtained as the four-quadrant arctangent phase angle from the phase space. Several methods have been developed for the calculation of the phase angles based on phase portrait analysis or the Hilbert transform [33,34,35]. Among those methodologies, the Hilbert transform-based method has been proved to be more robust than the phase angles in performing the phase portrait, especially regarding none-purely sinusoidal signals [33]. The Hilbert transform allows the transformation of any real signal into complex, analytic signal according to:

(2) ζ(t)=x(t)+iH(t)

at time *t_i_*, the phase angle can be computed by:

(3) $$\theta (t_{i})=\arctan\left(\frac{H(t_{i})}{x(t_{i})}\right)$$

The continuous relative phase CRP between two signals can be defined as the differences of the phase angles:

(4) $$CRP(t_{i})=\theta_{1}(t_{i})-\theta_{2}(t_{i})=\arctan\left(\frac{H_{1}(t_{i})x_{2}(t_{i})-H_{2}(t_{i})x_{1}(t_{i})}{x_{1}(t_{i})x_{2}(t_{i})+H_{1}(t_{i})H_{2}(t_{i})}\right)$$

where H_1_(t) and H_2_(t) are the Hilbert transform of signals of the proximal and distal segment, respectively. The CRP index can assume values between 0° and 180°. Values close to 0° indicate in-phase coupling of the two segments, while values close to 180° represent an out-phase coupling of signals.

To identify differences in inter-joint coordination between young and older adults, the mean absolute relative phase (MARP) was computed by averaging the absolute values of the curve points considering the overall trial duration [36], as in the following equation:

(5) $$\mathrm{MARP}=\frac{\sum_{i=1}^{p}|\bar{CRP_{i}}|}{p}$$

where *p* is the number of time points in each trial. Similar consideration done for the CRP can be adopted for the MARP value.

Furthermore, the Deviation Phase (DP) was analyzed in order to assess variability among trials in inter-joint coordination [36]. The DP can be assessed by averaging the standard deviation among trials of the CRP(*t_i_*) over the trial duration, as in the following equation:

(6) $$\mathrm{DP}=\frac{\sum_{i=1}^{p}SDi}{p}$$

where SDi represents the standard deviation of the CRP among the three trials at the *i*-th time instant. The DP is a measure of the stability organization provided by the neuromuscular system [36]. DP values close to 0 °C attest less intra-subject variability of the inter-joint coordination.

In order to assess postural control of body motion in the anterior-posterior and medio-lateral directions in response to external balance perturbations, body displacements of the head, the trunk and the pelvis were estimated via a strap-down integration of the acceleration signal, similarly to [37]. Body displacement of pelvis-sensor obtained through this method can be addressed as an estimation of the COM displacement, as authors reported [37]. However, differently from [37], in our study, we used the rotation matrix obtained from the quaternion output of the IMUs for the strapdown integration. In the original work [36] the authors rather used the gyro output for obtaining an estimation of the rotation matrix, as the system available for them did not provide the quaternion as an output.

To estimate the displacement, the acceleration signal was firstly rotated in the global coordinate frame to remove gravitational acceleration. After the gravitational acceleration removal, the acceleration signal of the inertial sensor in the global coordinate frame was straightforwardly integrated. Velocity was then high-pass filtered and the displacement was obtained through a second integration and filtering. The applied filters were zero-lag first-order Butterworth filters with a cut-off frequency of 0.2 Hz for the anterior-posterior (AP) and medio-lateral (ML) components and 0.5 Hz per the vertical (V) component.

Considering the body displacement, the following kinematic parameters were obtained for the statistical analysis: (i) the range of motion of the body segment displacement in the ML (RoM_ML_) and AP (RoM_AP_) directions expressed in mm; (ii) the total path length of the body displacement normalized to the task duration (PATH) and (iii) the maximum velocity of the displacement (MV) expressed in m/s. The before mentioned parameters were considered for the head, trunk, and pelvis displacements.

### 2.5. Statistical Analysis

All data were tested for normality by means of the Shapiro-Wilk test. Statistical analysis was performed with the SPSS package (IBM-SPSS Inc., Armonk, NY, USA). Two unpaired t-tests were conducted to assess differences in body mass and height between the two groups. In order to test differences in postural strategies induced by aging and different perturbation frequencies, a 2 × 2 two-way mixed ANOVA, with AGE as a between-subject factor (two levels: young adults and older adults) and FREQ (two levels: lower and higher frequency) as a within-subject factor was used separately for the EO and the EC conditions. When the assumption of sphericity was violated, the Greenhouse-Geisser correction was considered. A paired *t*-test within each group and an unpaired t-test between the two groups were performed when the interaction between the main effects was significant. The Bonferroni corrections were considered for all the statistical analysis. The significance level was set at 0.05 for all the statistical tests.

## 3. Results

All subjects were able to complete the experimental procedures without losing balance and/or experiencing fatigue.

No statistical differences were found in body mass and height between groups.

Considering all statistical analysis, the 2 × 2 two-way mixed ANOVA reported no significant interactions between main effects AGE and FREQ.

• G- and φ-absolute:

In Table 1, mean and standard deviation of G-absolute and φ-absolute values and *p*-values are reported.

**Figure 1:**
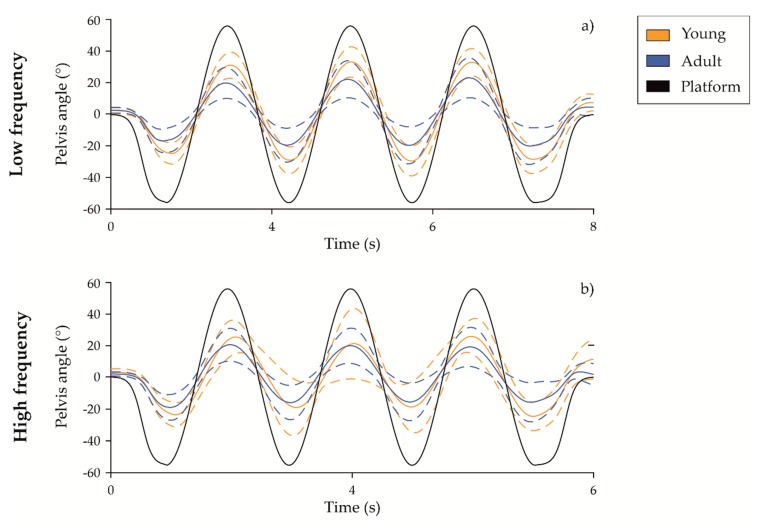
(**a**) Mean (solid line) and standard deviation (dashed lines) of pelvis angle in the transverse plane for the eyes opened condition during the low frequency task. (**b**) Mean (solid line) and standard deviation (dashed lines) of pelvis angle in the transverse plane for the eyes opened condition during the high frequency task. The orange curves refer to young group, the blue ones to the older adults while the black curve is platform trajectory.

With regards to AGE main effect in the EO task, older adults showed a significant lower G-absolute value of trunk and head than young adults. The pelvis body segment of young adults reached the highest mean values of G-absolute in both frequency conditions (57.04% and 48.00%), see Table 1 and Figure 1. Compared with younger population, older adults exhibited a smaller amount of the axial body motion. In the EC condition, a similar reduction of motion amplitude was found in G-absolute values of head and pelvis of older subjects. Considering the main effect FREQ, upper body G-absolute values statistically decreased as a function of rotation frequency increment, regardless of visual condition for all body segments.

Concerning AGE main effect, no differences were found in the EO condition related to φ-absolute. While the main effect of FREQ was found statistically significant for all the body segments. In the EC task, φ-absolute values of the pelvis were statistically different between young and older adults, according to AGE main effect. To face external yaw postural perturbations, older adults anticipated pelvis motion while younger adults adopted a delayed motion strategy. Head and pelvis phase shifts were found statistically significant in the main effect FREQ.

• G- and φ-relative:

In Table 2, mean and standard deviation of G-relative and φ-relative values and *p*-values are reported. In the EO task, the main effect AGE of the G-relative values were found to be statistically different for all the considered segment-couples. More specifically, young adults reported G-relative values close to 100% attesting a similar amplitude pattern between the rotation around the vertical axis of the proximal and the distal segment. By contrast, older adults exhibited lower values of G-relative attesting a reduction in amplitude pattern of the distal body compared to the proximal. As regard to the main effect FREQ, statically differences were found for all the considered body segment-couples. In the EC condition, similar trend of the EO condition was found for the main effect AGE. While regarding the main effect FREQ, statistical differences were found for the head-pelvis and trunk-pelvis couples, attesting that the amplitude value of the distal segment decreased as a function of frequency perturbation increment.

Regarding φ-relative, significant differences were found in the head-pelvis couple between young adults and older ones, in EO condition. More specifically, the distal segment of older adults appeared delayed with respect to the proximal one, while in the young group, synchronization strategies were adopted between segment couples. Increasing frequency motion, a higher delay was observed in the distal segment, especially in older adults.

• Continuous relative phase: MARP and DP:

In Table 3, mean and standard deviation of MARP and DP indices expressed in [°] are reported. In the EO task, age-related differences were found for all the segment-couples. Higher MARP values were reached by older populations who preferred a more anti-phase motion strategy in response to balance rotational disturbance. An in-phase coupling strategy was observed in the young subjects. Increasing perturbations intensity, similar in-phase motion strategy was observed for all segment-couples. Considering the EC condition, a similar trend was found both regarding AGE as main effect. While considering FREQ as main effect, a more anti-phase strategy was observed in both groups when increasing perturbation intensity.

**Figure 2:**
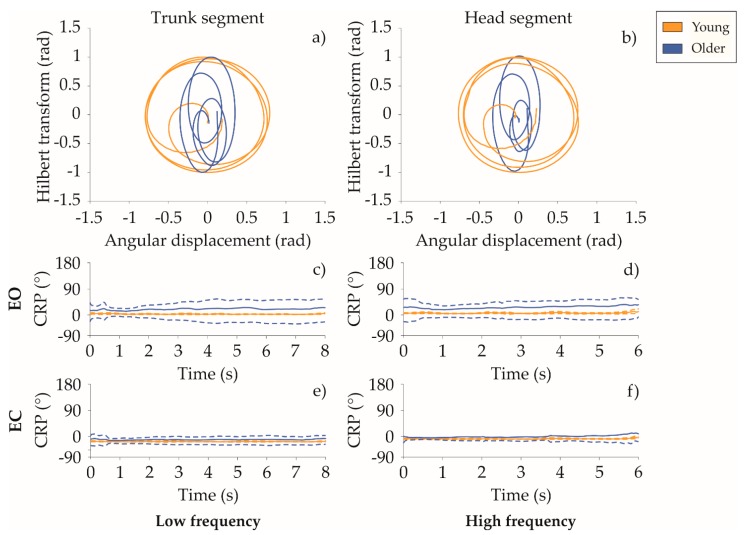
(**a**) and (**b**) are phase portraits of the trunk and head body segment of one healthy young subject and one older subject during EO condition at low frequency, respectively. (**c**–**f**) Mean (solid line) and standard deviation (dashed lines) of CRP of the head-trunk couple for the EO and EC condition for the low frequency task and EO and EC conditions for the high frequency task, respectively. The orange curves refer to the young group, the blue ones to older adults.

As regards coordination variability, older adults exhibited higher values of DP in all the segment-couples compared to young adults, regardless of the task condition (see Figure 2). Although the aforementioned results reported a wide amount of motor engagement, with the increase perturbation intensity, coordination variability of the trunk-pelvis couple increased as well in both visual conditions.

• Body displacement: RoM_ML_, RoM_AP_, PATH, and MV:

In Table 4, mean and standard deviation of upper body displacement measures and *p*-values of the mixed ANOVA are reported. In EO condition, RoM_ML_, PATH, and MV of the head and pelvis displacements were statistically lower in older adults compared to young adults. Regarding FREQ as a main effect, statistical differences were found considering PATH, MV in all upper body segments. Additionally, frequency increment effect was also found in RoM_ML_ and RoM_AP_ in trunk displacement. A similar trend was reported in the EC task for both main effects.

## 4. Discussion

In our study, age-related changes were found in balancing continuous yaw perturbations, regardless of visual condition and perturbation frequency. A more conservative and less destabilizing motion was observed as a postural strategy in the older population, suggesting that older adults compensate for their reduced physical capabilities by becoming more cautious while performing a postural task.

• G- and φ-absolute:

As the fast Fourier analysis on yaw segment angles reported, young adults showed a matching strategy between the body displacement and the platform rotation, taking into account the amplitude of the trajectories. Conversely, older adults exhibited a smaller amount of motion amplitude of the upper body to counteract an unbalanced rotational state. This reduction in amplitude was observed in all the upper body yaw angles in older adults, especially regarding the distal segments. As observed in our results, this stabilization strategy progressively decreases from proximal to distal segment, becoming noticeable especially in the head motion of the older adults when perturbation frequency increases. In this context, the different balance response of adults could be caused by different perceptions of the vestibular system. Perceiving risky head rotations, the vestibular system tends to modify body dynamics by minimizing head oscillations, regardless of visual information. Moreover, avoiding visual feedback, a delayed motor strategy of the proximal segment was found in comparison with the platform motion in the older population, attesting the incapability of older adults to manage perturbation with anticipatory adjustment of the axial body.

As reported in literature, continuous and predictable perturbations are more easily managed compared to discontinuous and impulsive perturbations [18]. However, contrarily to sudden perturbations, during continuous perturbation subjects blend two primarily postural mechanisms. Subjects focused on the adjustment of their body motion in accordance with the actual external perturbation [18]. Moreover, subjects anticipate the body motion predicting the mechanical effect of the reflexes triggered by the displacement itself. In this context, pursuing the external movement of the perturbation by complying balance response with the platform displacement was observed to be the most functional and less-expensive postural strategy adopted by healthy younger adults [14,18]. Similar postural strategies were observed in our results by healthy younger subjects in balancing continuous yaw perturbations.

• G- and φ-relative:

By considering the fast Fourier analysis on segment-couples, proximal and distal segments of the younger group were in perfect agreement regarding amplitude rotation as well as time shift. Conversely, older population motor response reported a reduction and a delay in distal segment rotation in comparison with the proximal one, especially considering the head-pelvis segment-couple. Basically, younger subjects were able to oscillate in accordance to platform motion. As a consequence, younger subjects adopted a strategy that minimized the active effort by generating a lower torque couple among body segment. Older adults instead reacted differently from young adults, adopting more complex motor control strategies targeted to head stabilization. These findings can be ascribed to the greater difficult of older subjects to manage head-trunk movements, as also demonstrated during walking tasks [38].

• Continuous relative phase: MARP and DP

As regards the coordination pattern, the CRP technique provides a measure of the coupling or the phase relationship between the actions of body segment-couples. Since coordination impairment represents a common sign among musculoskeletal [39] and neurological disease [40], the age-related differences in the coordinative compensatory strategy were investigated in this study. Segment-couple coordination patterns of the upper body were different in the two groups. During EO condition, compared to the older adults, the younger population responded coupling body segments across the trial and reporting a less variable intra-subject trial-to-trial relationship between the actions of the segment-couples. Older adults also tended to balance yaw perturbation moving joints in and out-of-phase opposite fashion with an increased variability among trials. These outcome can be justified by the well-known loss of spatio-temporal coupling of muscles during postural responses, as reported in [41].

Increased variability in coupling relationship due to the aging was already reported in Yen et al. [42] during the obstacle-crossing task. This age-related biomechanical modification is associated to a lower ability of older adults to maintain a stable body displacement when crossing obstacles with different heights. A similar trend was observed in our results when subjects shifted from eyes open to the eyes closed condition. The segment-couple coordination of the upper body assumed more in-phase motion behaviors. Avoiding out-phase segment movements might be a coordination strategy adopted in the process of mastering redundant degrees of freedom of the upper body in a more unsafe scenario.

**Figure 3:**
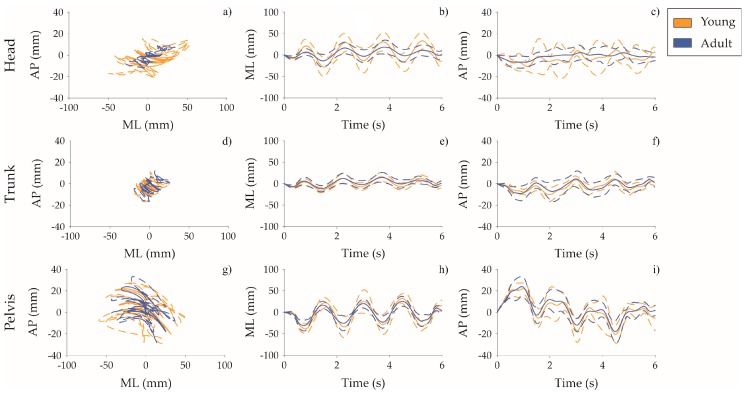
Mean (solid line) and standard deviation (dashed lines) of head, trunk, and pelvis displacement in the eyes opened condition during the high frequency task. The orange curves refer to the young group, the blue ones to older adults. In young group, medio-lateral sway of head is in phase opposition with respect to pelvis. Both have higher amplitude with respect to trunk, which acts as a pivot. This strategy is less evident in older subjects.

• Body displacement: RoM_ML_, RoM_AP_, PATH, and MV:

In terms of displacement of body segments, the analysis of the upper body unveiled that subjects behave as a double inverted pendulum, mainly in the frontal plane. As Figure 3 shows, both groups tended to assume a more oscillating displacement in the medio-lateral fluctuation of the head and the pelvis body segment with respect to the trunk. In particular, when visual information was allowed, head and pelvis medio-lateral movements were found to be both in a wider range, while the trunk stood noticeably more stable, acting as a center pivot. The upright balance was continuously reach by actively counteracting the head body segment with respect to the pelvis one, as they appear in almost perfect phase opposition (see Figure 3). In Figure 3, results of the eyes opened condition during the high frequency task were reported. However, a similar trend was observed in all the experimental tasks. Despite subjects were challenged with a rotational perturbation in the transversal plane, results highlighted motor behaviors similar to those of the double inverted pendulum already observed in previous studies, in which subjects were exposed to purely translational perturbations. This aspect could be justified considering the effect of the centrifugal force. In our interpretation, the rotational perturbation generated a centrifugal force, which tended to displace the body center of mass in medio-lateral direction. Subjects counteracted by translating head in the opposite direction. Head trajectory, in fact, appeared to be perfectly in opposition with respect to the pelvis one (see Figure 3), similarly to what happens due to the hip strategy in the case of translational perturbations [6].

Despite the before-mentioned trend reported in both groups, a prominent age-dependent stiffening strategy was witnessed by a smaller amount of medio-lateral oscillation, a lower path length and a lower mean velocity of the pelvis and head displacements. As previously reported in the literature, in the upright balance, humans can be sketched as an Acrobot [43], consisting in a series of inverted pendulums related to feet, legs and torso. When a large deflection of the base of support occurs, a quick shift of the COM is required to maintain the upright stance. In a human model, a proper displacement at the hip level modulates the torque created by the gravitational force on the shifted body, allowing the subject to keep the COM over the base of support and the foot flat on the ground [44]. By approximating the human torso as a single link, the head displacement acted in the opposite direction, allowing the generation of the counteractive moment of the gravitational head force.

Interestingly, a different motor strategy was observed when vision was denied. The absence of visual inflow changed the body motion strategy adopted to keep the balance. Since the hip strategy was apparently preserved, the head displacement in the sagittal plane appeared more evident, overshooting pelvis motion in both groups. As a consequence of visual condition, the counteraction of body inertia was mainly in charge of the somatosensory and vestibular reference control, which reported a less effective role compared to the visual reference control in maintaining head stabilization. Similarly to findings in Nardone et al. [14], in which subjects were exposed to continuous anterior-posterior external perturbation, head oscillations were found more prominent during eyes-closed tasks. On the contrary to [14], older adults stimulated by continuous yaw perturbation presented a lower amount of head displacement compared to younger population, both regarding the eyes-open and the eyes-closed conditions.

By summarizing, our results highlighted age-dependent differences in rotational, translational, and coupling motor behaviors of the upper body of subjects elicited by external yaw perturbations. Future developments could be focused on the design of rehabilitation programs targeted to restore those impaired motor strategies. Those programs will be beneficial in reducing the risk of fall in older adults, especially those occurring during turning tasks, which still represent the 13% of all real-life falls [45].

Although this study provides insight into contributing factors to manage unbalanced conditions, some limitations should be taken into account. The small sample size and the large variability in the phase shift parameters could have affected the statistical analysis biasing results. In a future study, it will be necessary to increase the number of the participants to enforce statistical results regarding age-related differences, and design rehabilitation program aimed to enhance motor deficit of older population.

## 5. Conclusions

In this paper, we addressed the question of how aging affects postural control by examining the kinematic response under external yaw perturbation. The fundamental age-related postural change was mainly observed in the head stabilization strategy. Outcomes of older adults reported a decreased amount of the rotational and the translational body motion with a tendency of delayed behaviors and an out-phase and highly-varied coordinative compensatory strategy of the upper segment-couples. During low-frequency perturbation, postural strategies implied an easier following-strategy of the platform motion reporting a more complex reflex response than an anticipatory postural adjustment. During a more demanding frequency yaw rotation, a counteractive motor response was observed by the stiffening of the upper body motion and by strongly acting on the body inertial control.

## Figures and Tables

**Table 1:** Mean and standard deviation of G-absolute values expressed as a percentage [%] and φ-absolute values expressed in [°] concerning head, trunk, and pelvis, for both lower (L) and higher (H) frequencies and visual conditions, i.e., eyes opened (EO) and eyes closed (EC) in young and older adults. *p*-values of the main effects, AGE and FREQ, of the two-way mixed ANOVA are reported. Statistical differences (*p* < 0.05) in the main effects are single-starred and reported in bold.

		EO-L		EO-H		*p*-Values		EC-L		EC-H		*p*-Values	
		Young Adults	Older Adults	Young Adults	Older Adults	AGE	FREQ	Young Adults	Older Adults	Young Adults	Older Adults	AGE	FREQ
**Head**	G	53.3 ± 19.4	21.5 ± 14.2	39.4 ± 16.1	15.6 ± 13.7	<0.01 *	<0.01 *	59.9 ± 21.2	41.2 ± 16.2	48.3 ± 17.8	25.2 ± 17.6	0.02 *	<0.01 *
	Φ	−4.8 ± 15.9	−1.6 ± 23.3	−25.3 ± 22.4	−18.5 ± 45.8	0.45	0.04 *	-13.8 ± 23.7	−2.4 ± 9.2	−25.4 ± 17.0	−13.3 ± 25.9	0.14	0.04 *
**Trunk**	G	55.2 ± 19.5	36.2 ± 18.3	45.0 ± 16.8	29.00 ± 21.2	0.04 *	<0.01 *	59.9 ± 21.1	47.2 ± 16.6	48.5 ± 17.1	30.0 ± 19.7	0.07	<0.01 *
	Φ	−3.7 ± 13.9	8.3 ± 19.3	−22.3 ± 22.3	5.8 ± 43.3	0.11	0.02 *	−12.6 ± 23.7	0.5 ± 8.9	−22.9 ± 16.6	−15.5 ± 35.8	0.24	0.06
**Pelvis**	G	57.0 ± 15.2	42.1 ± 14.5	48.0 ± 14.0	37.7 ± 16.8	0.09	<0.01 *	56.3 ± 15.3	48.2 ± 11.9	48.9 ± 13.9	39.0 ± 14.5	0.14	<0.01 *
	Φ	0.9 ± 9.8	6.00 ± 6.4	−7.7 ± 11.7	1.1 ± 9.7	0.10	<0.01 *	−2.1 ± 14.6	4.6 ± 6.0	−8.8 ± 11.2	3.6 ± 6.9	0.03 *	0.03 *

**Table 2:** Mean and standard deviation of G-relative values expressed as a percentage [%] and φ-relative values expressed in [°] concerning head-pelvis, trunk-pelvis, and head-trunk, for both lower (L) and higher (H) frequencies and both visual conditions, i.e., eyes opened (EO) and eyes closed (EC) in young and older adults. *p*-values of the main effects, AGE and FREQ, of the two-way mixed ANOVA are reported. Statistical differences (*p* < 0.05) in the main effects are single-starred and reported in bold.

		EO-L		EO-H		*p*-Values		EC-L		EC-H		*p*-Values	
		Young Adults	Older Adults	Young Adults	Older Adults	AGE	FREQ	YOUNG ADULTS	Older Adults	Young Adults	Older Adults	AGE	FREQ
**Head-Pelvis**	G	93.7 ± 17.8	47.0 ± 19.6	82.0 ± 23.9	39.5 ± 18.6	<0.01 *	<0.01 *	103.5 ± 18.0	83.0 ± 18.6	95.8 ± 18.3	61.2 ± 20.3	<0.01 *	<0.01 *
	Φ	−6.5 ± 9.2	−13.6 ± 35.3	−7.4 ± 12.9	−62.1 ± 54.0	**0.03**	<0.01 *	−10.7 ± 14.6	−20.4 ± 35.3	−15.4 ± 11.1	−27.8 ± 50.1	0.42	0.24
**Trunk-Pelvis**	G	101.4 ± 14.0	81.8 ± 17.9	93.3 ± 17.4	75.3 ± 23.8	0.02 *	0.02 *	103.1 ± 18.4	96.8 ± 13.1	97.0 ± 14.8	74.4 ± 20.5	**0.05**	<0.01 *
	Φ	−5.0 ± 6.2	−22.3 ± 59.2	−10.2 ± 9.0	−23.6 ± 37.7	0.32	0.46	−9.3 ± 13.7	−17.8 ± 37.4	−13.3 ± 10.4	−27.0 ± 46.4	0.41	0.12
**Head-Trunk**	G	91.7 ± 7.5	56.1 ± 16.6	85.4 ± 16.8	51.5 ± 17.4	<0.01 *	**0.04**	100.5 ± 1.9	84.8 ± 14.8	98.0 ± 6.5	82.5 ± 16.8	<0.01 *	0.12
	Φ	−1.5 ± 3.7	8.7 ± 35.0	2.9 ± 14.4	−38.5 ± 45.6	0.30	**0.03**	−1.4 ± 1.6	−2.5 ± 3.2	−2.1 ± 2.8	−0.8 ± 8.9	0.40	0.95

**Table 3:** Mean and standard deviation of the mean absolute relative phase (MARP) and Deviation Phase (DP) index of the continuous relative phase (CRP) technique, both expressed in [°] concerning head-pelvis, trunk-pelvis and head-trunk, for both lower (L) and higher (H) frequencies and both visual conditions, i.e., eyes opened (EO) and eyes closed (EC) in young and older adults. *p*-values of the main effects, AGE and FREQ, of the two-way mixed ANOVA are reported. Statistical differences (*p* < 0.05) in the main effects are single-starred and reported in bold.

			EO-L		EO-H		*p*-Values		EC-L		EC-H		*p*-Values	
			Young Adults	Older Adults	Young Adults	Older Adults	AGE	FREQ	Young Adults	Older Adults	Young Adults	Older Adults	AGE	FREQ
**Head-Pelvis**		MARP	6.7 ± 2.2	27.7 ± 22.1	15.2 ± 9.7	36.9 ± 20.7	<0.01 *	0.12	6.1 ± 1.8	14.3 ± 7.8	14.6 ± 8.7	33.9 ± 26.2	0.02 *	0.02 *
		DP	3.2 ± 1.4	23.4 ± 22.6	6.8 ± 5.4	23.9 ± 13.2	<0.01 *	0.57	3.8 ± 3.6	17.1 ± 20.6	5.6 ± 3.6	17.8 ± 9.9	0.02 *	0.77
**Trunk-Pelvis**		MARP	4.9 ± 2.5	26.5 ± 31.1	12.8 ± 10.4	32.1 ± 25.2	0.01 *	0.43	5.4 ± 2.1	7.9 ± 4.0	11.2 ± 6.8	33.7 ± 35.2	0.08	0.04 *
		DP	1.8 ± 0.8	11.6 ± 10.4	5.9 ± 4.1	20.1 ± 14.4	0.01 *	0.01 *	3.4 ± 3.3	7.9 ± 12.2	5.5 ± 4.3	21.6 ± 15.4	0.03 *	0.03 *
**Head-Trunk**		MARP	3.1 ± 1.4	21.4 ± 26.5	4.0 ± 1.3	34.0 ± 37.4	0.05 *	0.08	1.7 ± 0.7	5.9 ± 4.3	3.3 ± 1.4	13.5 ± 10.8	<0.01 *	0.07
		DP	1.8 ± 0.8	17.4 ± 19.4	2.3 ± 1.0	21.4 ± 21.05	0.02 *	0.43	1.1 ± 0.6	10.4 ± 15.2	1.6 ± 0.4	10.4 ± 6.6	**0.03**	0.92

**Table 4:** Mean and standard deviation of medio-lateral (ML) and anterior-posterior (AP) components of range of motion (RoM) expressed in [mm], the total path length of the body displacement divided by the task duration (PATH), and the maximum velocity of the displacement (MV) expressed in [m/s], all these displacement measures concerning head, trunk, and pelvis, for both lower (L) and higher (H) frequencies and both visual conditions, i.e., eyes opened (EO) and eyes closed (EC), in young and older adults. *p*-values of the main effects, AGE and FREQ, of the two-way mixed ANOVA are reported. Statistical differences (*p* < 0.05) in the main effects are single-starred and reported in bold.

		EO-L		EO-H		*p*-Values		EC-L		EC-H		*p*-Values	
		Young Adults	Older Adults	Young Adults	Older Adults	AGE	FREQ	Young Adults	Older Adults	Young Adults	Older Adults	AGE	FREQ
**Head**	RoM_ML_	88.5 ± 33.8	43.4 ± 16.6	83.5 ± 33.1	45.2 ± 28.4	0.01 *	0.08	109.5 ± 41.4	75.1 ± 34.0	117.8 ± 52.0	65.5 ± 37.2	0.04 *	0.29
	RoM_AP_	40.8 ± 15.8	24.1 ± 5.0	38.4 ± 17.9	23.9 ± 8.2	0.25	0.38	54.7 ± 22.6	36.0 ± 7.2	52.8 ± 24.1	40.1 ± 14.6	0.18	0.52
	PATH	0.4 ± 0.1	0.2 ± 0.1	0.4 ± 0.1	0.2 ± 0.1	0.02 *	<0.01 *	0.5 ± 0.2	0.3 ± 0.1	0.6 ± 0.3	0.3 ± 0.2	0.02 *	<0.01 *
	MV	0.3 ± 0.1	0.1 ± 0.1	0.3 ± 0.1	0.2 ± 0.1	0.02 *	<0.01 *	0.3 ± 0.2	0.2 ± 0.1	0.4 ± 0.1	0.2 ± 0.1	0.02 *	<0.01 *
**Trunk**	RoM_ML_	31.9 ± 9.0	28.2 ± 8.7	40.1 ± 8.6	34.7 ± 13.8	0.27	0.01 *	38.3 ± 12.6	34.2 ± 12.0	48.7 ± 18.8	41.5 ± 15.5	0.38	0.01 *
	RoM_AP_	20.9 ± 3.5	20.4 ± 5.3	23.6 ± 4.8	24.7 ± 10.4	0.88	0.03 *	24.4 ± 4.1	24.5 ± 5.5	27.6 ± 7.2	29.3 ± 9.5	0.75	0.01 *
	PATH	0.1 ± 0.1	0.1 ± 0.1	0.2 ± 0.1	0.2 ± 0.1	0.68	<0.01 *	0.2 ± 0.1	0.2 ± 0.1	0.2 ± 0.1	0.2 ± 0.1	0.52	<0.01 *
	MV	0.1 ± 0.1	0.1 ± 0.1	0.1 ± 0.1	0.1 ± 0.1	0.52	<0.01 *	0.1 ± 0.0	0.1 ± 0.0	0.2 ± 0.1	0.2 ± 0.1	0.38	<0.01 *
**Pelvis**	RoM_ML_	81.2 ± 25.7	57.0 ± 17.3	87.2 ± 20.1	63.2 ± 18.5	0.01 *	0.08	83.0 ± 15.4	65.2 ± 17.1	83.0 ± 25.4	47.4 ± 15.1	0.04 *	0.52
	RoM_AP_	52.1 ± 9.4	5.5 ± 3.0	52.9 ± 10.2	44.4 ± 15.0	0.25	0.38	52.6 ± 8.7	47.7 ± 14.9	57.5 ± 12.6	47.5 ± 15.1	0.18	0.29
	PATH	0.3 ± 0.1	0.2 ± 0.1	0.4 ± 0.1	0.3 ± 0.1	0.02 *	<0.01 *	0.4 ± 0.1	0.3 ± 0.1	0.4 ± 0.1	0.3 ± 0.1	0.02 *	<0.01 *
	MV	0.2 ± 0.1	0.2 ± 0.0	0.3 ± 0.1	0.2 ± 0.1	0.02 *	<0.01 *	0.2 ± 0.1	0.2 ± 0.0	0.3 ± 0.1	0.2 ± 0.1	0.02 *	<0.01 *
